# Review time of oncology drugs and its underlying factors: an exploration in China

**DOI:** 10.3389/fphar.2023.1151784

**Published:** 2023-11-01

**Authors:** Xingyue Zhu, Bao Liu

**Affiliations:** ^1^ Department of Pharmacy Administration, School of Medicine and Health Management, Guizhou Medical University, Guiyang, China; ^2^ Department of Health Economics, School of Public Health, Fudan University, Shanghai, China

**Keywords:** drug lag, review duration, drug approval, information vacuum period, quality of evidence

## Abstract

**Introduction:** How the launch delay of drugs and other factors of interest can influence the length of the review period by drug agencies is still unknown, and understanding this can help better strike the trade-off related to review speed.

**Methods:** We included all new oncology drug applications submitted to China’s National Medical Product Administration (NMPA) between 1 January 2018 and 31 December 2021, and ultimately succeeded in achieving marketing approval. For each drug, the length of the NMPA review process and other major characteristics were collected, including the registration class, approval class, priority review designation, and launch delay relative to the United States, as well as the number of patients enrolled, comparator, and primary endpoint of the pivotal trials supporting the approval. Linear regression model was employed to analyze the effects of factors of interest on the NMPA review time.

**Results:** From 2018 to 2021, NMPA received 137 oncology applications that were ultimately approved. Half of the approvals [76 (55.5%)] were first licensed in the US, leaving a median launch delay of 2.71 years (IQR, 1.03–5.59) in China. In the pivotal studies, the median enrollment was 361 participants (IQR, 131–682), and the use of control groups [90 (65.7%)] and surrogate endpoints [101 (73.7%)] was prevalent. The median review time was 304 days (IQR, 253–376). Multivariate analysis for log-transformed review time showed that larger enrollment ( > 92) was associated with a drop of 20.55% in review time (coefficient = −0.230; 95% CI, −0.404 to −0.055; *p* = 0.010); and a short delay (0 < delay ≤ 1.95 years) was associated with a drop of 17.63% in review time (coefficient = −0.194; 95% CI, −0.325 to −0.062; *p* = 0.004).

**Discussion:** The short launch delay relative to the US was one important driver to the review speed of NMPA, which might suggest its latent regulatory reliance on the other global regulator during the post-marketing period when new information on the drug’s clinical benefit was still lacking.

## 1 Introduction

The primary objectives of a drug agency are to evaluate the efficacy and safety profiles of pharmaceuticals and to provide prompt access to new drugs, of which the benefits outweigh the risks. To measure the performance of drug agencies, it is important to consider two aspects, namely, the number of “right” decisions made, which means that an approved drug is effective and is not withdrawn from the market due to safety issues after approval, and the duration required for these decisions to be reached. A lengthy review process will prevent patients from timely health improvement and discourage future investment in pharmaceutical development (Philipson et al., 2008; Vernon et al., 2009; Chorniy et al., 2021). However, it can also enhance the scrutiny of submitted material so as to raise the probability of arriving at the “right” decisions (Olson, 2008; Carpenter et al., 2012). This is the well-known trade-off between benefit and risk (Grabowski and Vernon, 1983).

In different regions, this trade-off is affected by distinct factors. In China, the oncology pharmaceutical market has experienced rapid growth, attracting both domestic and foreign developers to deliver their innovative products to Chinese patients as quickly as possible (Lythgoe et al., 2023a; Zhang et al., 2023). However, there persists a significant delay in the launch of new drugs in China compared to the United States (US) or the European Union (EU) (Zhu and Liu, 2020; Li and Yang, 2021). Luo et al. (2023a) documented that less than half of the oncology drugs approved by the United States Food and Drug Administration (FDA) in 2010–2021 were marketed in China, and the median time of launch delay for the mutually approved drugs decreased from 5.4 years in 2010–2015 to 2.7 years in 2016–2021. The trend and causes of launch delay have been the focus of the scientific community, while the influence of delay receives less discussion. We assume that the launch delay would have an impact on the drug review process of a regulator in the delayed region. On one hand, the first global approval of a drug by the FDA or European Medicines Agency (EMA) can act as a signal of its acceptable drug profiles and facilitate its subsequent approval in other countries. As the most influential drug authorities, the FDA and EMA lead global industry standards and guidance, and are known for their strict and sound drug review procedures. Many smaller national medicine regulators (e.g., Caribbean and Latin American) even follow their regulatory decision-making directly (Durán et al., 2021). On the other hand, the launch delay can provide a time gap, allowing for further evidence generation (Kashoki et al., 2020), and it will take time to re-judge the risk–benefit balance of a drug. In particular, for new drugs approved based on a surrogate endpoint, which are exemplified by drugs receiving the FDA’s accelerated approval designation (U.S. Food and Drug Administration, 2018), the time gap avails the validation of the surrogate endpoint in delayed regions. However, when the innovative drug is submitted in other areas immediately after its initial global approval, there may be little new evidence. In such a situation, the launch delay will be a vacuum of information, and the subsequent agencies may find reassurance from the decisions made by the first regulator, especially those by the FDA or EMA. It is still unknown whether new drugs with launch delay will undergo a review procedure of different lengths. Moreover, since 2015, China has rolled out a series of reforms in its drug regulation system, including the inception of the priority review pathway in 2016 (Yao et al., 2017; Li et al., 2021). This pathway aims to abbreviate the stipulated review duration from 200 to 130 days for drugs with significant clinical benefits or those addressing urgent medical needs (Li et al., 2021). More details on the expedited pathways in China and a comparison with their major international analogs are given in Supplementary Table S1. The priority review pathway is expected to affect the review time.

Other factors relevant to the delicate trade-off between benefit and risk are also of concern. Ideally, the drug’s clinical benefit, which refers to the treatment effect on a patient’s survival, function, or feeling, should be established via well-designed randomized controlled trials that typically require large enrollment. This course to demonstrate the clinical benefit is definitely costly and time-consuming. Thus, surrogate endpoints have gained popularity in drug clinical development to expedite access to promising new drugs as substitutes for clinical benefit (Chen et al., 2019; Gyawali et al., 2023). The FDA defines a surrogate endpoint as a bio-marker or laboratory measure that is reasonably likely to predict the clinical benefit but cannot measure the clinical benefit itself (U.S. Food and Drug Administration, 2018). For example, the objective response rate and progression-free survival (PFS) are frequently used as surrogacy for overall survival (OS) in the oncology area (Sacher et al., 2014; Kemp and Prasad, 2017). Since 2017, surrogate endpoints have been allowed to form the regulatory basis for China’s new expedited pathway, conditional approval, which is analogous to the FDA’s accelerated approval (Supplementary Table S1). Nonetheless, the use of surrogate endpoints in drug development has raised criticism as well (Booth et al., 2023). The lax standards for surrogate endpoints have been called into question since many used surrogate measures are unvalidated or have been found only to have a poor correlation with clinical benefit (Kim and Prasad, 2016; Walia et al., 2022). In China, 24.1% of new oncology approvals did not demonstrate substantial improvements in OS, notwithstanding the gain on surrogate measures (Zhang et al., 2022). Apart from surrogate endpoints, single-arm trials are likewise increasingly prevalent in drug development (Luo et al., 2023b; Huang et al., 2023; Ribeiro et al., 2023). The single-arm design, coupled with surrogate endpoints, can enroll small numbers of patients (Burzykowski et al., 2005; Gyawali et al., 2023), which accommodates orphan drugs suffering from limited patient pools (Tenhunen et al., 2020). However, in March 2023, the FDA issued a draft guidance encouraging the use of surrogate endpoints in randomized controlled trials, rather than in single-arm studies, to support accelerated approval (Benjamin and Lythgoe, 2023; U.S. Food and Drug Administration, 2023); in that, some surrogacies in single-arm trials, like the response rates, can further add uncertainty to the assessment of the safety and/or effectiveness of a drug (Gyawali et al., 2023; U.S. Food and Drug Administration, 2023). This draft presents the regulator’s concern about drug benefit uncertainty stemming from trial designs. Whether pivotal trials with different design features influence the performance of a regulatory agency in terms of the speed of drug evaluation and review still awaits to be answered. It is plausible that well-controlled trials serving as the gold standard for regulatory approval can provide clear evidence and facilitate the scientific review. In contrast, when uncertain or lower-quality evidence is obtained from single-arm, small-scale trials with surrogate endpoints, the ambiguity in the relationship between the surrogates and the real OS benefits can be pretty challenging for decision-making.

This study makes the first attempt to investigate the factors of review time based on oncology drugs approved by China’s National Medical Product Administration (NMPA) and specifically explores the potential effects of drug launch delay. Our work will conduce to further understanding of the decision-making of the drug regulatory agency and build the base for to-be research and policies to strike the inherent trade-off in drug approval.

## 2 Materials and methods

### 2.1 Data

We used the marketed drug database of the Center of Drug Evaluation (CDE) (NMPA Center of Drug Evaluation, 2022), which is subordinated to NMPA and responsible for drug review, to identify all oncology approvals of new molecular entities and biologics, including their initial marketing approvals and new indication supplements. The backlog of applications used to be significant in CDE, and the resultant queuing time brought about extremely lengthy review duration (Zhou et al., 2017). Since the reforms started in 2015, the backlog issue has been gradually addressed. In 2018, it was reported that > 90% of applications were delivered decisions within the stipulated timeframe (NMPA Center of Drug Evaluation, 2018). Hence, only approvals that were submitted between 1 January 2018 and 31 December 2021 were included.

**FIGURE 1:**
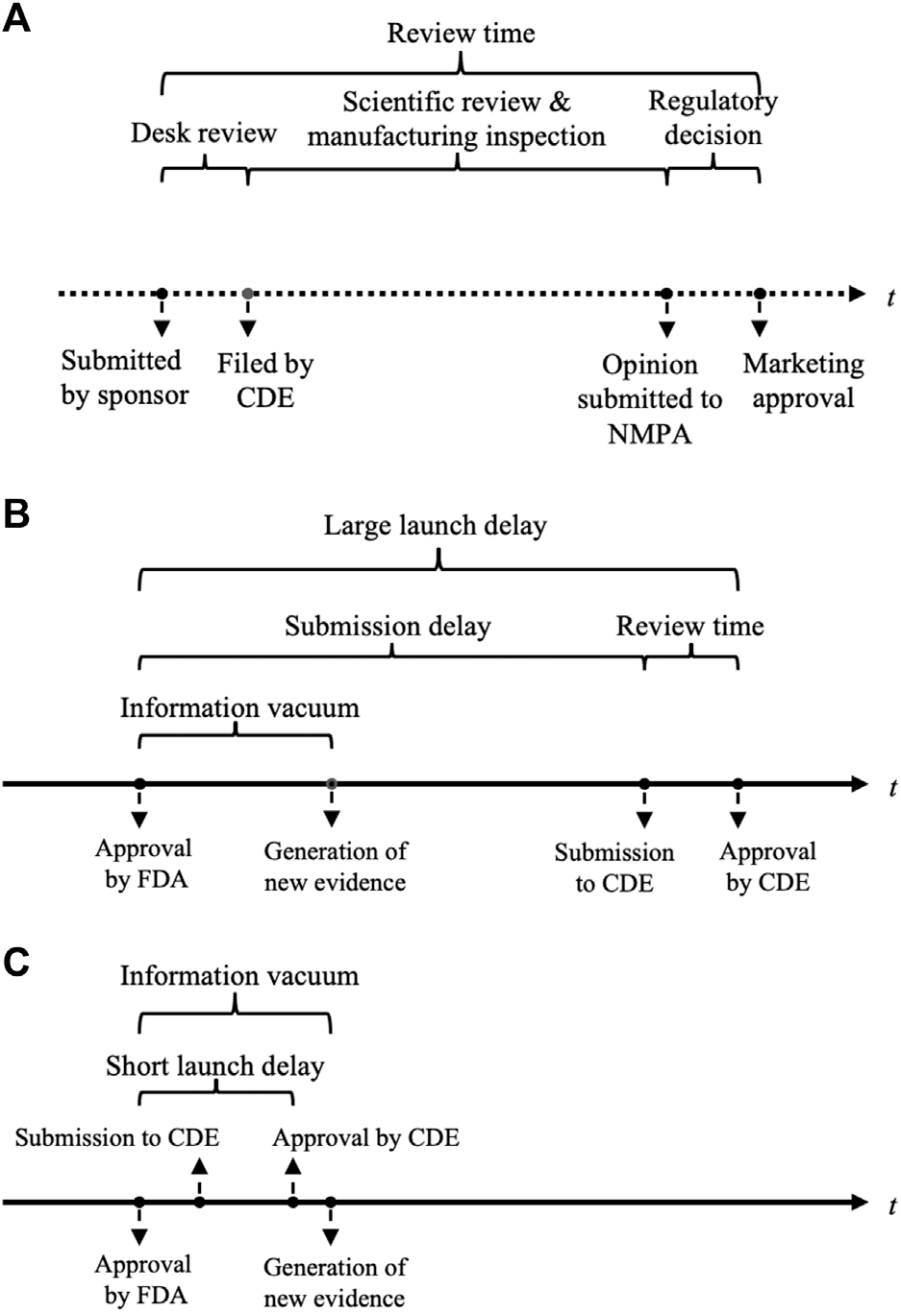
Illustrations of the drug review process in China and launch delay between the United States and China. **(A)** Process of drug review and evaluation in NMPA. **(B)** A large launch delay allows new evidence to be generated and analyzed. **(C)** A short launch delay can fall within the information vacuum period after a drug’s first global approval and leaves little time for new evidence. NMPA, China National Medical Product Administration; FDA, the United States Food and Drug Administration; CDE, China Center of Drug Evaluation.

The NMPA’s drug review process is shown in Figure 1A. Review time was defined as the time gap from the submission date of a new drug application (NDA) or biologics license application (BLA) to the date of marketing authorization. For each approval, the disclosed CDE review report provided information on the review time, and the key characteristics of registration class (NDA or BLA), approval class (initial marketing or new indication supplement), sponsor nationality, cancer type, priority review designation, as well as pivotal trial(s) features of the number of enrolled participants, primary endpoints, and control groups. If the approval was supported by more than one pivotal trial, information about the comparator and primary endpoints was extracted from the first (randomized) trial, while the enrollment size was the sum of all pivotal studies. If the drug had obtained approval in the US before being marketed in China, the delay between the approval timings in China and the US was measured. The drug submission and approval dates in the US were derived from Drugs@FDA. The composition of launch delay is illustrated in Figures 1B, C. Primary endpoints were grouped into three classes: the first was OS, as long as OS was listed as one of the primary endpoints; the second was surrogate endpoints related to survival, including PFS, disease-free survival, relapse-free survival, and metastatic-free survival; and the third was surrogate endpoints related to the response rate, including the objective response rate and other measures based on the response rate.

### 2.2 Statistical analysis

Descriptive statistics were used to characterize the included approvals. The *t*-test and ordinary least square (OLS) regression were used for univariate and multivariate analyses. The dependent variable, review time, was significantly right-skewed and, therefore, underwent natural log transformation. The primary eight covariates were included in the OLS model: registration class, approval class, priority review designation, approved year, launch delay, enrollment, control groups, and primary endpoint class. It should be noted that, to clarify the effects of some numeric variables, categorization was needed. The existing literature has found that single-arm phase 2 trials (Ladanie et al., 2020), or surrogate endpoints (Downing et al., 2014; Puthumana et al., 2018), typically enroll smaller numbers of patients. As such, we assumed that the number of patients enrolled in a trial could serve as a proxy for trial design, and a small enrollment could imply that the design was less likely to demonstrate the true benefit–risk balance of a drug. Accordingly, maximally selected rank statistics were applied to enrollment size to find its optimal cut-off value for defining the small enrollment, resulting in a calculated cut-off value of 92. Thus, we defined the enrollment size ≤ 92 as the small enrollment in our study. The length of launch delay was another variable that needed to be categorized. In the multivariate analysis, the length of launch delay would not help explain the variance in review time. This was mainly attributed to the fact that drugs first approved in China had no delay (delay = 0), and a large number of these drugs would lead to an extreme right skew in the length of launch delay. In this case, to categorize launch delay would be informative. Similar dichotomization was hence performed to the length of launch delay to define a short delay, and a dummy variable was created with an estimated optimal cut-off value of 1.95 years: 0 indicated that the first approval was in China, ≤ 1.95 years indicated a short delay, and > 1.95 years indicated a large delay. For the sake of parsimony, a backward stepwise procedure was used to select variables. Furthermore, to take into account the potential concern of endogeneity of launch delay, a two-stage least square (2SLS) approach was utilized with the FDA review time and development strategy (domestic, overseas, and out-licensed) as instruments. A significance level of 0.05 was set for two-tailed tests, and a robust standard error was employed. Stata version 15 (StataCorp LP) and R version 4.0.2 (maxstat package) were used to conduct the analysis.

## 3 Results

### 3.1 Features of the included approvals

**FIGURE 2:**
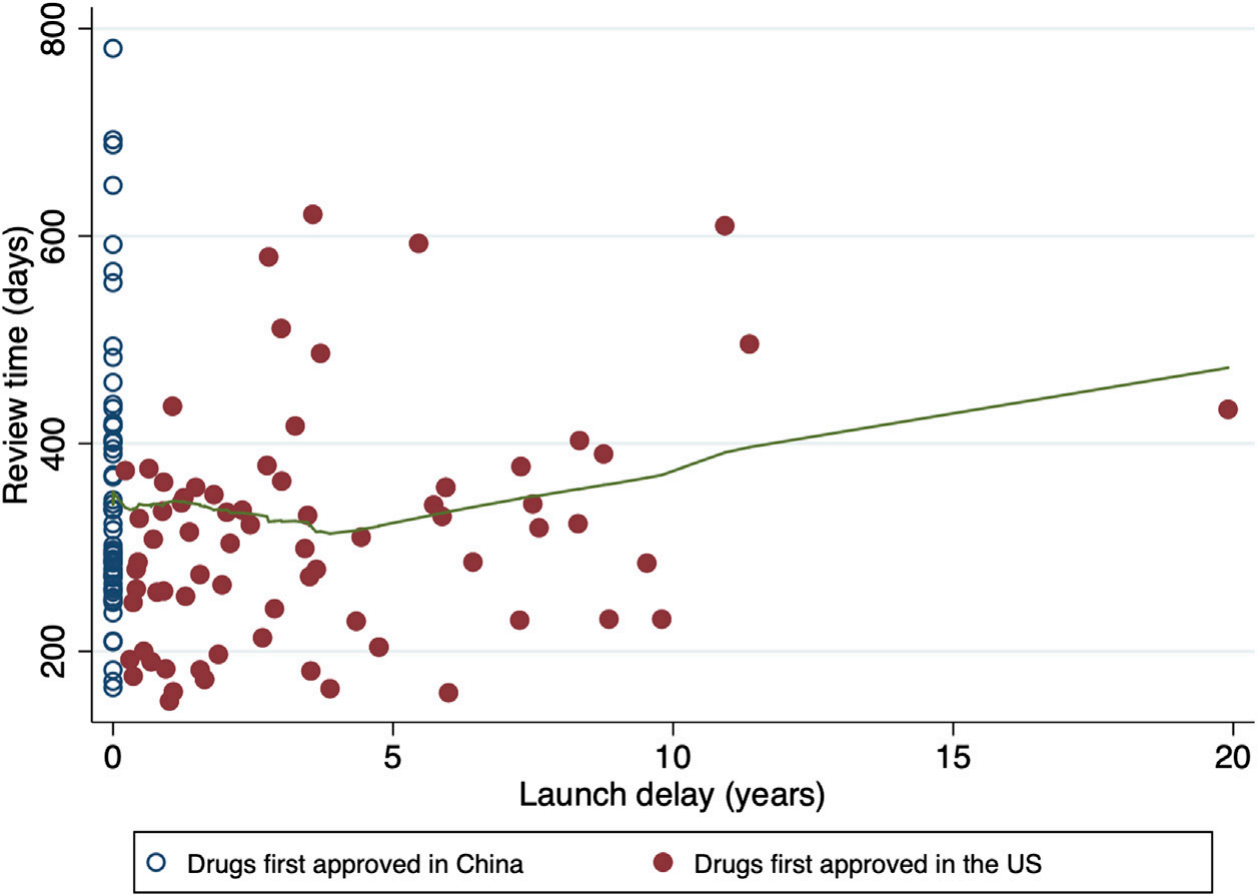
Distribution of review time in terms of launch delay. Hollow circles denote the drugs first approved in China, while solid circles denote the drugs first approved in the US. The fitted line in green is constructed by the locally weighted scatterplot smoothing (LOWESS) approach, which indicates the tendency of review time.

From 1 January 2018 through 31 December 2021, a total of 137 oncology applications were submitted to and ultimately approved by NMPA (Table 1). The median review time for all the included approvals were 304 days (IQR, 253–376). New molecular entities and biologics each accounted for half of the total (50.4% *versus* 49.6%). There were more new indication supplements [77 (56.2%)] than marketing approvals [60 (43.8%)]. In terms of therapeutic areas, the most common cancer types were lung cancer [33 (24.1%)] and lymphoma [17 (12.4%)]. A priority review pathway was granted to most approvals [98 (71.5%)]. Less than half of the drugs were first approved in China (61[44.5%]), and they were all developped by Chinese pharma companies. The remaining 76 approvals (55.5%) had been licensed in the US before being marketed in China, leaving a median launch delay of 2.71 years (IQR, 1.03–5.59). The maximum delay was observed for yttrium-90 microsphere (SIR-SPHERES^®^), at a remarkable duration of 19.91 years. The distribution of review time in terms of the length of launch delay is shown in Figure 2, where the underlying trend indicated the slightly shortened review duration for approvals with a launch delay < 3 years. In the pivotal trials supporting these approvals, the median enrollment was 361 participants (IQR, 131–682), and the small enrollment ( ≤ 92) made up 13.9%. One-third of the trials did not include a comparator group [47 (34.3%)]. The clinical endpoint of OS was not frequent [36 (26.3%)], while surrogate endpoints related to survival [49 (35.8%)] or the response rate [52 (38.0%)] dominated in the pivotal trials.

**TABLE 1:** Characteristics of included approvals and their pivotal studies.

Characteristic	N (%)
**Approval-related**	
Registration class	
NDA	69 (50.4)
BLA	68 (49.6)
Approval class	
Marketing	60 (43.8)
Supplement	77 (56.2)
Cancer type	
Lung cancer	33 (24.1)
Lymphoma	17 (12.4)
Breast cancer	10 (7.3)
Genitourinary cancer	9 (6.6)
Leukemia	8 (5.8)
Liver cancer	8 (5.8)
Head and neck cancer	7 (5.1)
Skin cancer	7 (5.1)
Gynecological cancer	6 (4.4)
Myeloma	5 (3.6)
Esophageal cancer	4 (2.9)
Thyroid carcinoma	4 (2.9)
Gastric cancer	4 (2.9)
Microsatellite instability-high tumors	4 (2.9)
Gastrointestinal stromal tumor	2 (1.5)
Pleural mesothelioma	2 (1.5)
Neuroendocrine neoplasm	2 (1.5)
Other	5 (3.6)
Priority review designation	98 (71.5)
Launch delay (n = 76), years, median (IQR)	2.71 (1.03–5.59)
Class of launch delay	
0	61 (44.5)
> 0 and ≤ 1.95 years	33 (24.1)
> 1.95 years	43 (31.4)
Review time, days, median (IQR)	304 (253–376)
Approved year	
**Pivotal trial-related**	
Enrollment size, median (IQR)	361 (131–682)
≤ 92	19 (13.9)
> 92	118 (86.1)
Control groups	
Yes	90 (65.7)
No	47 (34.3)
Class of primary endpoint	
OS	36 (26.3)
Surrogate endpoints related to survival	49 (35.8)
Surrogate endpoints related to the response rate	52 (38.0)

NDA, new drug application; BLA, biologics license application; IQR, interquartile range; OS, overall survival.

### 3.2 Factors on review time

**FIGURE 3:**
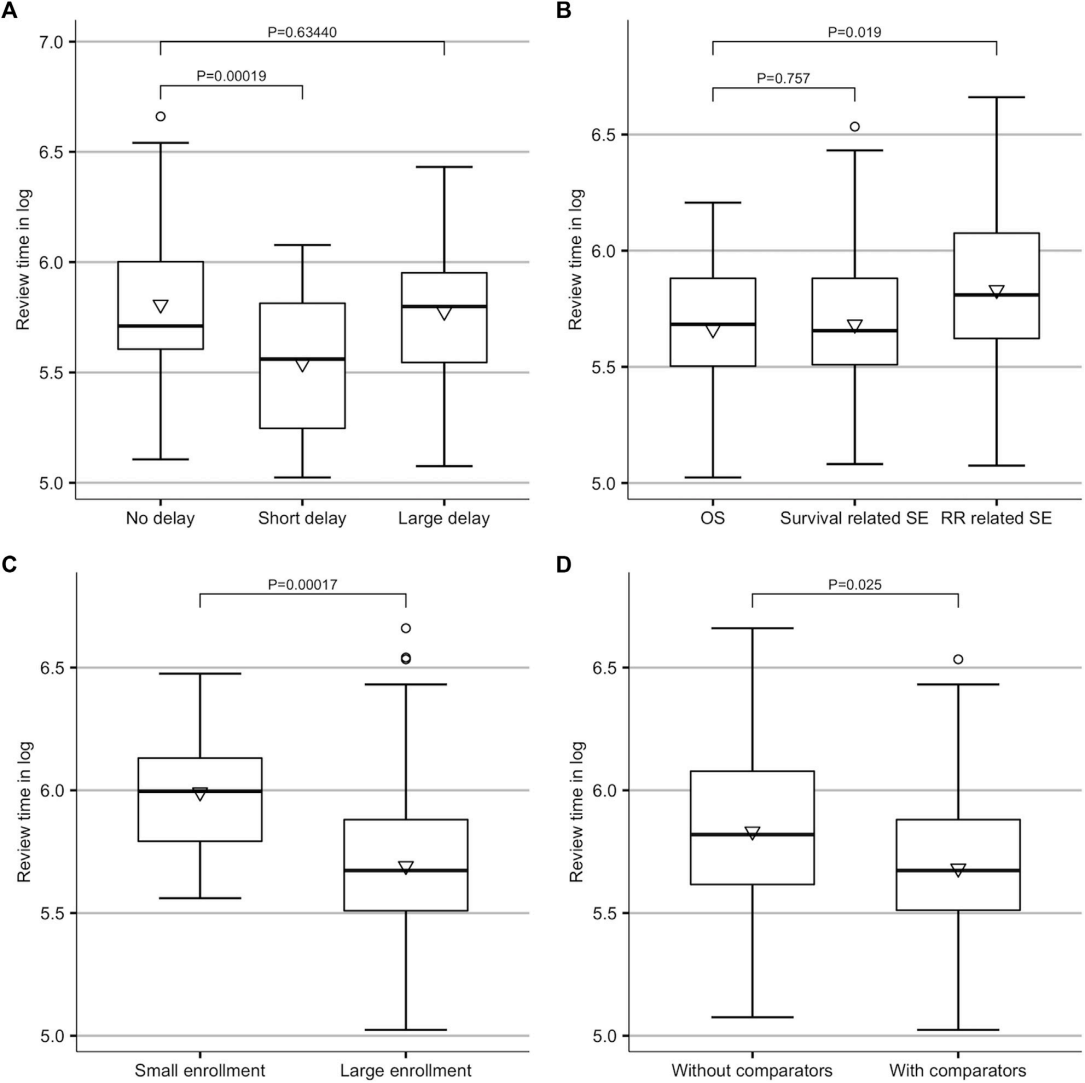
Results of univariate analysis. **(A)** Difference amongst no delay (delay = 0), short delay (0 < delay ≤ 1.95 years), and large delay (delay > 1.95 years). **(B)** Difference amongst OS, survival-related surrogate endpoint, and response rate-related surrogate endpoint. **(C)** Difference between small enrollment (enrollment ≤ 92) and large enrollment (enrollment > 92). **(D)** Difference between with and without control groups. The whiskers indicated the error bars, the middle line indicated the median, the inverted triangle indicated the mean, and the circle indicated outliers. OS, overall survival; SE, surrogate endpoint; RR, response rate.

A univariate analysis was conducted for the class of delay and other major factors (Figure 3 and Supplementary Table S2). When compared to approvals with no launch delay, a short delay ( > 0, and ≤ 1.95 years) seemed to be associated with a reduced review duration (*p* < 0.001), while a significant association was not observed for a large delay ( > 1.95 years) (*p* = 0.634). Surrogate endpoints related to the response rate appeared to correlate with longer review time (*p* = 0.019), but the difference between the surrogates related to survival and OS was not significant (*p* = 0.757). A larger enrollment size ( > 92) (*p* < 0.001) and control groups (*p* = 0.025) were both linked to significantly shorter review time.

The results of the multivariate analysis are presented in Table 2. When compared to those first approved in China, drugs that were first approved in the US and only experienced a short launch delay had an increased review speed by 17.63% (coefficient = −0.194; 95% CI, −0.325 to −0.062; *p* = 0.004), but there was no significant change in review time for drugs with longer delays. As to the features of the pivotal trials, the expansion of the enrollment size was associated with a drop of 20.55% in review time (coefficient = −0.230; 95% CI, −0.404 to −0.055; *p* = 0.010). No significant difference was observed between trials with and without control groups. The review time did not vary across drugs with the different classes of endpoints as well. New indication supplement approvals had a faster review process than marketing approvals (coefficient = −0.240; 95% CI, −0.363 to −0.117; *p* < 0.001). The priority review pathway significantly improved the review speed by 19.35% compared to the standard review (coefficient = −0.215; 95% CI, −0.332 to −0.098; *p* < 0.001). Registration class was not correlated with the review time. The time trend of review duration was not significant, implying a steady review speed throughout the study period. The stepwise procedure resulted in selecting the class of launch delay, enrollment size, approval class, and priority review as the major explanatory factors (Supplementary Table S2), suggesting the robustness of the results from the OLS model.

**TABLE 2:** Effects of factors of interests on the review time.

Variable	OLS		2SLS	
	Coefficient (95% CI)	*p*-value	Coefficient (95% CI)	*p*-value
Enrollment size				
≤ 92	0 [Reference]		0 [Reference]	
> 92	−0.230 (−0.404 to −0.055)	0.010	−0.244 (−0.415 to −0.073)	0.005
Control groups				
No	0 [Reference]		0 [Reference]	
Yes	0.037 (−0.199–0.273)	0.758	−0.009 (−0.254 to 0.236)	0.941
Class of primary endpoint				
OS	0 [Reference]		0 [Reference]	
SE related to survival	−0.050 (−0.175 to 0.076)	0.436	−0.061 (−0.187 to 0.064)	0.338
SE related to RR	0.017 (−0.182–0.215)	0.869	−0.081 (−0.301 to 0.139)	0.472
Class of launch delay				
0	0 [Reference]		0 [Reference]	
> 0, and ≤ 1.95 years	−0.194 (−0.325 to −0.062)	0.004	−0.357 (−0.578 to −0.136)	0.002
> 1.95 years	−0.056 (−0.200 to 0.089)	0.449	0.026 (−0.133–0.185)	0.748
Registration class				
BLA	0 [Reference]		0 [Reference]	
NDA	0.060 (−0.061–0.182)	0.327	0.061 (−0.055–0.176)	0.305
Approval class				
Marketing	0 [Reference]		0 [Reference]	
Supplement	−0.240 (−0.363 to −0.117)	< 0.001	−0.211 (−0.335 to −0.087)	0.001
Priority review				
No	0 [Reference]		0 [Reference]	
Yes	−0.215 (−0.332 to −0.098)	< 0.001	−0.166 (−0.289 to −0.043)	0.008
Approved year	−0.041 (−0.104 to 0.022)	0.198	−0.038 (−0.101 to 0.025)	0.235
N	137		136	
*R* ^2^	0.3137		0.2421	

OLS, ordinary least square; 2SLS, two-stage least square; OS, overall survival; SE, surrogate endpoint; RR, response rate; NDA, new drug application; BLA, biologics license application.

Review time constitutes the total launch delay (Figure 1), and the lengthy review process is an important driver of drug delay issue. Hence, one may be concerned about the endogeneity of launch delay. A 2SLS model was applied to deal with this reverse causal effect, and the results are listed in Table 2. The short delay remained to correlate with a faster review duration, with a larger effect size than the OLS model (coefficient = −0.357; 95% CI, −0.578 to −0.136; *p* = 0.002). This finding implied that the magnitude of the effect of launch delay on review speed may be discounted if we did not consider the causal dependency. The effects of enrollment, approval class, and priority review remained significant. The 2SLS model helped us alleviate the endogeneity problem.

## 4 Discussion

This study contributed to the existing body of the literature on the issue of new drug launch delay. We found that the gap between launch timings in China and the US acted as a predictor for the NMPA’s faster review procedure. It was interesting that, nonetheless, the finding implied that only drugs with a short launch delay (for instance, less than 1.95 years) were likely to be reviewed more quickly, whereas a longer delay did not seem to exert a significant influence on the review time. One potential explanation is that, during the short time gap, subsequent regulatory agencies depend on the same evidence package used by the initial regulator to evaluate the drug application as new data about efficacy and safety are being generated and readouts are still very early. For example, the median time from drug approval to the occurrences of the first safety event and the first serious event was 1.75 and 2.31 years, respectively (Zhu and Liu, 2022). In this vacuum period, where there is no more learning about the drug’s profiles (as shown in Figure 1), the marketing authorization by the world’s largest drug regulator is itself a plausible signal of acceptable uncertainty about the drug’s actual benefit. Consequently, it may, to some extent, facilitate regulatory approval by other drug agencies, particularly for serious conditions with urgent unmet needs. This finding may indicate a latent form of regulatory reliance. The World Health Organization defines regulatory reliance as “the decision-making of the national regulatory authority in one jurisdiction may take into account and give significant weight to assessments performed by another national authority or trusted institution” (World Health Organization, 2020). The direct recognitions of the FDA-approved drugs in some Latin American countries (Durán et al., 2021) and the recent proposal in the United Kingdom for “near automatic sign off” for drugs that have been approved by trusted regulators (Lythgoe and Sullivan, 2023) are typical regulatory reliance. In contrast, the magnitude of the NMPA’s trust in the actions of the FDA is much less, but it can still have an influence on the vacuum period of drug information. As the launch delay prolongs, further evidence for new drugs emerges, and new data are still uncertain to prove favorable. As such, a thorough review will be required to evaluate the updated drug profiles after the information vacuum period, and the effect of reliance will diminish. Future research is expected to determine the length of the vacuum period.

The latent regulatory reliance can help the earlier entry of new drugs into the market but without impacting the true benefit. Actions during the information vacuum period are important to redress the balance between speed and risk. For drugs that experience launch delays and are ultimately approved under high uncertainty, post-marketing studies with robust designs should be required to validate the clinical benefit. In addition, given that data processing and publication often take time, relying on open sources to obtain post-first-approval drug information may lengthen the vacuum period. NMPA will benefit from establishing an information-sharing mechanism with its major international counterparts. This would allow for the timely utilization of raw data generated in regions where the drug has already been introduced, enabling NMPA to make informed decisions and helping minimize this vacuum period.

The FDA and EMA are also recognized as benchmarks for other national authority agencies in terms of performance evaluation. Regarding the review speed for oncology drugs, we found that NMPA [304 days (IQR, 253–376)] was slower than FDA [median 200 days (IQR, 155–277)] but faster than EMA [426 days (IQR, 358–480)] (Lythgoe et al., 2022). This result indicates that NMPA can perform close to its international counterparts and demonstrates the Chinese regulator’s efforts to reduce redundancy in the review process. However, the disparity between NMPA and FDA or EMA is still in place: the approval concordance rate between FDA and EMA exceeds 90% (Kashoki et al., 2020), while we found that only 55.5% of approvals by NMPA had been authorized in the US. This is partly caused by the development strategies of pharmaceuticals and the difference in clinical evidence facing the agencies. FDA and EMA frequently have a substantial overlap in received drug applications and have the same dataset for review and evaluation (Kashoki et al., 2020). However, for NMPA, the evidence package provided to FDA/EMA may not be reflective of Chinese populations and *vice versa* (Benjamin et al., 2022). Recently, sintilimab, an innovative checkpoint inhibitor developed in China, was refused by FDA despite its significant affordability (Singh et al., 2022). One important reason was that the evidence exclusively from Chinese patients underrepresented the US population (Singh et al., 2022). The reverse delay of Chinese new drugs in other regions may raise some concerns (Barrios et al., 2023), particularly for low- and middle-income countries that rely on the FDA’s decisions (Lythgoe and Sullivan, 2022). To reduce the access gap, coordination of drug development and approval decision-making between China and the US and EU is of much value, e.g., to establish a concurrent drug review system between China and these regions, similar to the Project Orbis (Lythgoe et al., 2023b; U.S. Food and Drug Administration. FDA, 2023) but which also necessitates huge cross-regional endeavors.

This study also examined the effects of the pivotal trial design features on the length of the drug review process. We found that an expanded enrollment of pivotal trials could save nearly 20% of the time spent in the review process. Small enrollment commonly represents the less-rounded research design, such as surrogate endpoints in single-arm trials. This design would perform inferiorly to confirm the real added benefit of new drug candidates. In addition, the safety profiles are usually under-characterized in small samples. Admittedly, a trial sample size is supposed to be determined according to the power analysis that ensures an adequate number of subjects to answer the primary study questions. In accordance with prespecified statistical requirements, small samples can produce reliable estimates of surrogate endpoints with scientific significance but which do not necessarily indicate sufficient clinical relevance concerning OS or the quality of life. To ascertain the risk–benefit balance based on the very limited data is still challenging. Moreover, small samples may also be a sign of rare diseases. In these diseases, clinical trials tend to have smaller enrollment and more flexible designs to ensure feasibility, even at the cost of increased uncertainty in the obtained evidence (Cheng et al., 2012; Korchagina et al., 2019).

Surrogate endpoints appeared to have no considerable effect on the review time after controlling for the effect of trial size. It should be noted that all the approvals with the small enrollment in our study had surrogate measures as the primary endpoint, and the strength of correlation for the same surrogate endpoint can vary across tumor types, treatment types, and lines of therapy (Gyawali et al., 2020). As such, one potential explanation is that the small enrollment is not only a sign of rare diseases but also indicates the attendant lack of surrogate endpoint validation in these diseases. In less rare conditions, more knowledge about the strength of surrogacy has been acquired. Then, the agency can use the surrogate as the basis for regulatory approval with enhanced confidence. However, in rare/ultra-rare conditions, the correlations between the real benefits and the observed effects on the surrogate are not yet well-understood. Consequently, the great uncertainty may preclude a fast review. NMPA can issue guidance on trial designs in drug development, based on its acceptance of uncertainty about drug benefits, to inform the industry and facilitate fast and safe access to new drugs.

Supplement approvals went through a significantly shorter review process. This result was mainly due to the reduced workload needed in reviewing new additional evidence for the same drug. Regarding new indication supplements, the emphasis of the review is primarily on efficacy profiles, while evaluations on pharmacokinetics/pharmacodynamics and safety can be saved a lot. Additionally, as the pathway that curtails the timeframe of the review process, priority review directly results in a faster evaluation of drug applications, which is confirmed in this study.

The previous study also reported that the difference in staffing and team efficiency across review divisions in a drug agency caused disparities in review time between therapeutic classes (Tabarrok et al., 2014). However, this is not the case for CDE since it has a much simpler structure. There are three divisions responsible for scientific review: Division 1, responsible for evaluating oncology products of both chemical entities and biologics; Division 2, which does not involve oncology products; and Biologic Division, responsible for assessing vaccines, regenerative therapies, and gene therapies. Most of our study drugs were expected to be evaluated by the same team. Hence, staffing and team efficiency will not raise confounding.

Certain conditions may have distinguishing features that influence the time CDE takes to complete the review. Nevertheless, among oncology therapies, we argued that the prevalence of a disease would hold greater significance in determining the complexity of the review, as compared to the specific cancer type. Orphan drugs may introduce additional challenges to the review process as less scientific knowledge about rare diseases has been accumulated. However, the CDE does not grant orphan designation, so we could not capture the effects of different disease categories.

We have discussed further limitations to our study as follows: first, our results did not reflect causal effects. All the review reports for oncology drugs that had been disclosed by CDE during the study period were collected, but there are some drug documents that have not yet been disclosed. It was unclear whether these disclosed ones were representative of the total. In addition, before the formal review procedure, many sponsors would meet CDE to discuss scientific details about obtained data or future development plans, which are known as pre-IND meetings, end-of-phase 2 meetings, and pre-NDA meetings. Communications between the reviewer team members and sponsors allow identifying key issues at early stages and offer opportunities for interactions to find solutions, which may save some time both in drug development and scientific review. However, information about the meetings was frequently absent in the review reports. On this account, confounding of pre-meetings might be introduced. The cut-off values of enrollment and the length of delay used in this study were data-driven, and one should be very cautious to extrapolate them to other contexts. At last, the therapeutic value of drugs is a major determinator of regulatory decision-making. Further studies should delve into the effects of therapeutic value on review time and drug launch delay.

## 5 Conclusion

We provided the first exploration of the factors in review time of drug agency based on data from China’s NMPA, which observed that a short delay was conducive to a higher review speed, but a longer delay seemed irrelevant. Our analysis presented the preliminary evidence of the drug information vacuum period and the NMPA’s latent regulatory reliance on the existing approvals by the other agencies. More efforts are needed for the drug authority to better strike the trade-off between reducing uncertainties and promoting the availability of promising new drugs.

## Data Availability

The raw data supporting the conclusion of this article will be made available by the authors, without undue reservation.
