# Supplementary material for: Review time of oncology drugs and its underlying factors: an exploration in China
Source: Front Pharmacol. 2023 Nov 1;14:1151784. doi: 10.3389/fphar.2023.1151784 (PMC10654631; doi:10.3389/fphar.2023.1151784)
Supplement: Supplementary file 1 [file Table1.DOCX]

Supplementary Material

Review Time of Oncology Drugs and its Underlying Factors: An Exploration in China

**Xingyue ZHU^1,^****^†,*^, Bao LIU^2,^****^†,*^**

***Correspondence:**

Bao LIU, [liub@fudan.edu.cn](mailto:liub@fudan.edu.cn)

Xingyue ZHU, [zhuxingyue@gmc.edu.cn](mailto:zhuxingyue@gmc.edu.cn)

# Supplementary Tables

Supplementary Table 1. Introductions of expedited review pathways in China.

| Expedited pathways | Established year | Stage | Scope | Measures |
| --- | --- | --- | --- | --- |
| **China NMPA: priority review** | 2015 | Drug review | Drugs with significant clinical benefits. | Abbreviate the review timeframe from 200 working days (for standard review) to 130 working days. |
| The US FDA: priority review | 1992 |  | Drugs would be significant improvements in the safety or effectiveness. | Abbreviate the review timeframe from 10 months (for standard review) to 6 months. |
| The EU EMA: accelerated assessment | 2005 |  | medicinal product is expected to be of major public health interest, particularly from the point of view of therapeutic innovation. | Reduce the timeframe to 150 days (compared to 210 days for standard review) |
| **NMPA: conditional approval** | 2017 | R&D | Innovative medications indicating for seriously debilitating or life-threatening conditions. | Approval based on surrogate endpoints, an intermediate clinical endpoint or investigational clinical data. Post-approval confirmatory trials that verify clinical benefit are mandatory. |
| FDA: accelerated approval | 1992 |  | Drugs for serious conditions that filled an unmet medical need. | Approval based on surrogate endpoints or an intermediate clinical endpoint. Post-approval confirmatory trials that verify clinical benefit are mandatory. |
| EMA: conditional market authorization | 2006 |  | Medicines that address unmet medical needs. | Approval based on less comprehensive clinical data than normally required, where the benefit outweighs the risk. It requires ongoing or new studies or collecting additional data to confirm the medicine's benefit-risk balance remains positive. |
| **NMPA:** **breakthrough therapy** | 2020 | R&D | Drugs for serious or life-threatening diseases and with preliminary clinical evidence that suggests substantial improvement over existing therapies. | Receive more intensive guidance from NMPA; eligibility for priority review designation and rolling submission. |
| FDA: breakthrough therapy | 2012 |  | Drugs that are intended to treat a serious condition and preliminary clinical evidence indicates that the drug may demonstrate substantial improvement over available therapy on a clinically significant endpoint. | Intensive guidance from FDA on an efficient drug development program, beginning as early as Phase 1; organizational commitment involving senior managers; all benefits of fast track designation. |
| EMA: PRIME (priority medicines) | 2016 |  | Medicines that target an unmet medical need and provide any available data showing a meaningful improvement of clinical outcomes. | Enhanced interaction and early dialogue between EMA and developers of promising medicines, to optimize development plans and speed up evaluation so these medicines can reach patients earlier; eligibility for accelerated assessment. |

Supplementary Table 2. Results of univariate analysis.

| Variable | Coefficient (95% CI) | P value |
| --- | --- | --- |
| **Class of launch delay** |  |  |
| 0 | 0[Reference] |  |
| $>$0, and $\leq$1.95 years | -0.267  (-0.401 to -0.133) | <0.001 |
| $>$1.95 years | -0.032  (-0.167 to 0.102) | 0.634 |
| **Class of primary endpoint** |  |  |
| OS | 0[Reference] |  |
| SE related to survival | 0.021  (-0.112 to 0.154) | 0.756 |
| SE related to RR | 0.169  (0.018 to 0.310) | 0.029 |
| **Enrollment size** |  |  |
| $\leq$92 | 0[Reference] |  |
| $>$92 | -0.300  (-0.434 to -0.165) | <0.001 |
| **Control groups** |  |  |
| No | 0[Reference] |  |
| Yes | -0.151  (-0.281 to -0.021) | 0.023 |

Supplementary Table 3. Results of the stepwise linear regression.

| Variable | Coefficient | Robust SE | P value | 95% CI |
| --- | --- | --- | --- | --- |
| Enrollment size |  |  |  |  |
| $\leq$92 | 0[Reference] |  |  |  |
| $>$92 | -0.218 | 0.080 | 0.007 | -0.376 to -0.059 |
| Class of launch delay |  |  |  |  |
| 0 | 0[Reference] |  |  |  |
| $>$0, and $\leq$1.95 years | -0.174 | 0.064 | 0.007 | -0.299 to -0.048 |
| $>$1.95 years | -0.035 | 0.065 | 0.592 | -0.164 to 0.094 |
| Registration class |  |  |  |  |
| BLA | 0[Reference] |  |  |  |
| NDA | -0.257 | 0.057 | <0.001 | -0.370 to -0.144 |
| Priority review |  |  |  |  |
| No | 0[Reference] |  |  |  |
| Yes | -0.182 | 0.047 | <0.001 | -0.275 to -0.090 |
